# PT-TDGCN: Pre-Trained Trend-Aware Dynamic Graph Convolutional Network for Traffic Flow Prediction

**DOI:** 10.3390/s25216709

**Published:** 2025-11-03

**Authors:** Hanqing Yang, Sen Wei, Yuanqing Wang

**Affiliations:** Department of Traffic Engineering, College of Transportation Engineering, Chang’an University, Xi’an 710064, China; 2019021093@chd.edu.cn (H.Y.); weisen@chd.edu.cn (S.W.)

**Keywords:** traffic flow prediction, pre-training, dynamic graph, convolutional trend-aware attention

## Abstract

Accurate traffic flow prediction is vital for intelligent transportation systems, yet strong spatiotemporal coupling and multi-scale dynamics make modelling difficult. Existing methods often rely on static adjacency and short input windows, limiting adaptation to time-varying spatial relations and long-term patterns. To address these issues, we propose the Pre-trained Trend-aware Dynamic Graph Convolutional Network (PT-TDGCN), a two-stage framework. In the pre-training stage, a Transformer-based masked autoencoder learns segment-level temporal representations from historical sequences. In the prediction stage, three designs are integrated: (1) dynamic graph learning parameterized by tensor decomposition; (2) convolutional trend-aware attention that adds 1D convolutions to capture local trends while preserving global context; and (3) spatial graph convolution combined with lightweight fusion projection for aligning pre-trained, spatial, and temporal representations. Extensive experiments on four real-world datasets demonstrated that PT-TDGCN consistently outperformed 14 baseline models, achieving superior predictive accuracy and robustness.

## 1. Introduction

With the rapid evolution of information and communication technologies, urbanization has accelerated worldwide, leading to concentrated populations and travel demand in large- and medium-sized cities. This growth has intensified congestion, accident risks, and resource allocation pressures, posing unprecedented challenges to urban traffic management systems. In this context, intelligent transportation systems (ITSs) have emerged as a critical infrastructure for smart cities, where accurate traffic state prediction serves as a cornerstone for applications such as congestion mitigation through route guidance and tidal control, refined signal timing and capacity allocation, real-time order dispatching for mobility platforms, and emergency response to extreme weather or incidents [1,2]. However, traffic data are inherently nonlinear, noisy, non-stationary, and heterogeneous, while simultaneously exhibiting multi-scale variations—daily and weekly cycles, long-term trends, short-term perturbations, and abrupt anomalies—making precise characterization of their complex spatiotemporal dependencies highly challenging [3].

Over the past decades, extensive studies have been conducted on traffic prediction. Early methods relied on statistical and traditional machine learning approaches such as Historical Average (HA) [4], Vector AutoRegression (VAR) [5], AutoRegressive Integrated Moving Average (ARIMA) [6], Support Vector Regression (SVR) [7], and Artificial Neural Network (ANN) [8]. These methods offer interpretability and computational efficiency but often depend on linear assumptions and overlook spatial correlations, thus underperforming when handling nonlinear, heterogeneous, and time-varying interactions. The rise of deep learning has spurred the development of neural network-based prediction frameworks [9]. RNNs and their variants excel in capturing sequential temporal dependencies [10,11,12], while CNNs have been employed to learn spatial correlations within neighboring regions [13,14,15,16]. Nevertheless, CNN-based methods are constrained by Euclidean grid assumptions [17], which fail to represent the complex non-Euclidean topology of real-world road networks. To address this limitation, graph neural networks (GNNs) have been widely adopted to model road networks as graphs, enabling direct representation of spatiotemporal dependencies in non-Euclidean spaces [18,19,20,21,22]. Representative works such as STGCN [23], DCRNN [24], GWNET [25], STSGCN [26], and STFGNN [27] have significantly improved accuracy and robustness by integrating graph convolution and attention mechanisms.

Despite these advances, GNN-based approaches still face three major challenges. First, many rely on distance, connectivity, or handcrafted features to construct static graphs. Such predefined structures fail to adapt to real-world dynamics, where commuting peaks, road closures, accidents, or extreme weather can rapidly reshape spatial dependencies. Although adaptive graphs have been proposed, learning interpretable and sparse dynamic adjacency matrices in large, noisy, and heterogeneous networks remains difficult. Second, mainstream temporal relies on RNN-based recursive propagation or CNN-based sliding convolutions. RNNs suffer from gradient vanishing and memory decay, while CNNs are limited by fixed receptive fields, making them better at capturing local short-term dependencies than long-range or cross-window temporal correlations. Real traffic, however, contains multi-scale phenomena-daily/weekly/holiday cycles, slow trends, and sudden shocks—where distant upstream events can impact downstream conditions after a delay. Models focusing only on nearby time slices may misalign or misinterpret global temporal semantics, leading to errors during peak transitions or mode shifts. Third, many frameworks predict using only 12 recent time steps (e.g., one hour), which stabilizes training but discards rich long-term history. Nighttime stable periods, for example, provide limited information for forecasting sharp morning surges. Conversely, incorporating the entire long history introduces redundancy, computational overhead, and overfitting risks. Thus, efficiently extracting salient long-term features while filtering noise and redundancy remains an open problem.

To address these challenges, we propose a novel framework, the Pre-trained Trend-aware Dynamic Graph Convolutional Network (PT-TDGCN), which integrates a pre-trained stage, dynamic graph learning, and convolutional trend-aware attention to enhance both prediction accuracy and robustness. The key contributions of this study are summarized as follows:

1. We proposed PT-TDGCN for spatiotemporal traffic flow prediction, structured around pre-trained and prediction paradigm. In the pre-trained stage, a Transformer-based masked autoencoder performs segment-based temporal feature modelling on extended historical sequences, explicitly capturing long-term dependencies and contextual semantics. In the prediction stage, the latest subsequence is encoded and combined with spatiotemporal modules for precise forecasting;
2. We designed a tensor decomposition based dynamic graph learner that adaptively models time-varying spatial dependencies across intervals and integrates with graph convolution in an end-to-end manner, enhancing adaptability and representational power;
3. We introduced 1D convolution into the projection of multi-head self-attention to encode local trend patterns while preserving global dependencies. This mechanism alleviates mismatches caused by similar values but divergent trends, improving robustness and phase alignment under anomalies or abrupt changes.
4. We conducted experiments on four real-world datasets, comparing PT-TDGCN with 14 baseline models. Results demonstrated consistent improvements across three metrics, validating the framework’s effectiveness and adaptability in complex traffic prediction scenarios

## 2. Related Work

### 2.1. Traffic Flow Prediction

In recent years, Graph Neural Networks (GNNs) excel at modelling non-Euclidean dependencies and have achieved strong results across relational domains such as social networks and knowledge graphs [28,29]. Coupling GNNs with temporal learners has advanced the study of spatiotemporal correlations by jointly capturing spatial topology and temporal dynamics [30,31,32,33]. The growing availability of graph-structured traffic data has also enabled the transfer of convolutional operators into the graph domain, fostering Graph Convolutional Networks (GCNs) and accelerating graph-based methods in engineering applications.

Spatiotemporal GNNs (STGNNs) typically integrate a graph module with a sequential model and can be grouped into three lines. (1) RNN-based approaches (e.g., DCRNN [24], AGCRN [34], TGCN [20]) propagate hidden states over time while aggregating spatial features via graph convolution. They model short-term variations well but suffer from high computational cost, gradient vanishing, and memory decay, which hinder learning multi-day or weekly dependencies. (2) CNN-based approaches (e.g., STGCN [23], GWNET [25], LSGCN [35], STSGCN [26], STFGNN [27]) employ 1D or temporal convolutions—often dilated or multi-scale—to parallelize temporal modelling and expand the receptive field, offering a better efficiency–accuracy trade-off than RNNs, yet with a still-bounded effective context. (3) Transformer-based approaches (e.g., GMAN [36], ASTGCN [37], STTN [38], TFormer [39]) use self-attention to establish global temporal dependencies, which are combined with spatial attention or graph operators to capture long-range interactions and complex dynamics.

Despite these advances, two limitations persist. First, many methods rely on predefined adjacency to encode relationships among road segments. Such fixed structures poorly reflect time-varying spatial couplings and congestion propagation under non-stationary conditions, creating structural rigidity. Second, to control computation, models are often trained and evaluated on short historical windows, which restricts the effective receptive field of attention or convolution and impedes the extraction of periodicity and long-term trends. Simply extending the input length greatly increases memory and runtime, complicating robust long-horizon modelling and reliable extrapolation under abrupt changes.

More recent hybrids explicitly learn dynamic adjacency. DGCRN [40] updates the graph jointly with recurrent states and DSTAGNN [41] alternating staged spatiotemporal aggregation with attention to refine time-varying connectivity. While these approaches demonstrate the potential of combining dynamic graphs with attention mechanisms, they typically depend on short supervised windows and do not incorporate an explicit contextual temporal prior derived from long historical sequences.

In summary, balancing computational feasibility with the ability to jointly capture dynamic spatial dependencies, long-term temporal correlations, and short- to medium-term trend features remains a central challenge and developmental direction for spatiotemporal graph modelling. To address this, researchers have actively explored strategies such as adaptive dynamic graph construction, long-sequence representation learning, and pre-trained paradigms, aiming to enhance the expressiveness, generalization, and practical applicability of models under complex and non-stationary traffic patterns.

### 2.2. Pre-Training Method

Pre-training has become a central driver of multimodal learning: models first acquire transferable representations from large-scale, task-agnostic data and are then adapted to downstream tasks via lightweight fine-tuning [42]. In natural language processing (NLP), this paradigm has been exemplified by two representative models: BERT [43], which employs a Transformer encoder backbone to construct bidirectional contextual representations and achieves outstanding performance on discriminative tasks; and GPT [44], which is based on a Transformer decoder and excels at the autoregressive generation of coherent and contextually relevant sequences. Both frameworks share the Transformer architecture, which, due to its efficient self-attention–based sequence modelling capability, has become the de facto standard for large-scale pre-trained models [45].

In vision, images are reformulated as patch sequences, enabling ViT [46] and BEiT [47]. Among them, Masked Autoencoders (MAEs) [48] introduce a mask-and-reconstruct paradigm that hides most patches, encodes visible, and reconstructs masked content to learn strong features under high masking ratios. This idea extends to TST [49] and other modalities, including Point-MAE [50] and VideoMAE [51]. For spatiotemporal data, TFormer [39] applies MAEs to generate intermediate representations that enhance the predictive performance of graph neural networks. Similarly, ExtraMAE [52] tailors masking for RNN backbones, and Ti-MAE [53] and LSTTN [54] leverage the Transformer’s capacity for long-range structure to design pre-training frameworks for forecasting, underscoring the MAE family’s versatility for long-term dependency modelling.

Motivated by these advances, we incorporated a dedicated pre-training stage that leverages Transformer-based self-attention with a customized masking strategy. The design explicitly models temporal regularities and contextual dependencies in ultra-long historical sequences, producing transferable subsequence-level representations. These representations strengthen long-term and contextual encoding, substantially improving downstream accuracy and robustness, while reducing reliance on handcrafted priors and mitigating challenges from data scarcity and distribution shifts. Consequently, our approach provides a generalizable MAE-grounded pre-training paradigm with potential applicability across diverse multimodal spatiotemporal prediction scenarios.

In addition, this departs from representative Transformer–GNN hybrids such as GMAN, TFormer, and TSFormer, which do not employ a dedicated masked pre-training stage and typically adopt a single-stream attention-graph pipeline without our tensor-decomposition–parameterized dynamic graph learner or convolutional trend-aware attention.

## 3. Method

### 3.1. Preliminary

Traffic road networks constitute the fundamental infrastructure of transportation systems, where individual road segments (or sensors) are interconnected to collectively support the overall operational framework. To formally represent such structured relationships in modelling, the road network is typically expressed as a topological graph G=(V,E,A). Here, $V=(v_{1},v_{2},\ldots ,v_{N})$ denotes the set of nodes corresponding to N road segments or monitoring points, $E=\left\{e_{ij}\right\}$ represents the set of directed edges, and $e_{ij}$ indicates the directed connection from node i to node j. The adjacency matrix A is used to characterize the connectivity strength between nodes.

The objective of traffic flow prediction is to accurately infer future traffic states by leveraging the spatiotemporal dependencies embedded within historical observations, under the constraint of a known road network topology. Formally, let the input sequence be defined as $v_{T}=\left\{X_{1},X_{2},\ldots ,X_{T}\right\}$, and the target output sequence as $v_{P}=\left\{X_{T+1},X_{T+2},\ldots ,X_{T+P}\right\}$. The prediction task can thus be formulated as learning a mapping function $f\left(\cdot \right)$:

(1) $$\left\{X_{T+1},X_{T+2},\ldots ,X_{T+P}\right\}=f\left(\left\{X_{1},X_{2},\ldots ,X_{T}\right\},G\right)$$

### 3.2. The Framework of PT-TDGCN

**Figure 1:**
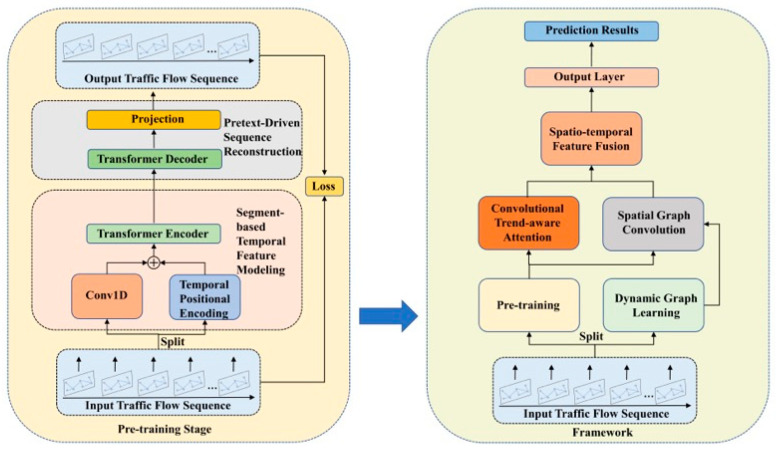
The Framework of PT-TDGCN.

This study introduces PT-TDGCN, a two-stage framework for traffic flow prediction that targets complex spatiotemporal dependencies, as shown in Figure 1. In the pre-training stage, extended historical sequences are processed by a segment-based Transformer encoder with 1D-convolutional embeddings and temporal positional encodings. A masked reconstruction pretext task learns context-rich subsequence representations and strengthens long-range temporal dependencies for transfer to downstream forecasting. In the prediction stage, the most recent subsequence is encoded by the pre-trained encoder and combined with an adaptive spatial module and a trend-aware temporal module. Spatially, a dynamic graph learner parameterized by tensor decomposition produces time-varying adjacency, on which graph convolution aggregates information from local neighbors and long-range connections. Temporally, a convolutional trend-aware attention integrates 1D-convolutional projections with multi-head self-attention to encode local trend variations while preserving global dependencies, reducing mismatches between similar magnitudes with divergent trends. A lightweight fusion then concatenates subsequence, spatial, and temporal features, followed by an MLP for multi-step prediction. Compared with prior Transformer–GNN hybrids, the design focuses on three essential differences: a dedicated masked pre-training stage on ultra-long histories, a tensor-decomposition dynamic graph for adaptive spatial structure, and convolution-augmented attention for explicit trend modelling. Together, these components deliver accurate and robust forecasting under diverse, non-stationary scenarios.

### 3.3. Pre-Training

The pre-training stage is the first step and targets ultra-long historical sequences to learn context-rich subsequence embeddings that transfer directly to forecasting. Unlike prior Transformer–GNN hybrids that train end-to-end without a dedicated pre-training phase, we employed a Transformer-based masked autoencoder so the encoder first internalizes long-range temporal regularities before fine-tuning. We jointly used subsequence reconstruction and a self-supervised prediction signal to strengthen trend awareness and improve robustness under distribution shifts, providing a stronger initialization than training from scratch. This design focused on what differs essentially from existing approaches: a stand-alone pre-training stage aligned with time-series structure and tailored to capture long-term context for downstream GNN-augmented forecasting. The pre-training process consists of two main modules: (1) Segment-based Temporal Feature Modelling, which explicitly encodes temporal dependencies across subsequences; and (2) Pretext-driven Sequence Reconstruction, which restores masked subsequences to optimize contextual feature learning.

#### 3.3.1. Segment-Based Temporal Feature Modelling

When modelling long traffic flow sequences, directly learning fine-grained representations at each individual time step is often inefficient and prone to redundancy in local information. To enhance both the representational capacity and computational efficiency of temporal modelling, we adopted a subsequence masking and reconstruction strategy. Specifically, the long historical sequence was divided into several short, non-overlapping subsequence units, and contextual information was leveraged to generate subsequence-level representations that explicitly encode trend features.

Following the approaches in [39,53,54], the original historical sequence $X_{hist}=\left[X_{t-L},X_{t-L+1},\ldots ,X_{t-1}\right]$ is segmented along the temporal axis into non-overlapping M subsequences, each of length s, resulting in a total of M=L/s subsequences, where L denotes the length of the input long sequence. For each subsequence, a one-dimensional convolutional layer (1D Convolution) is first employed to extract local temporal patterns as the initial embedding representation. Subsequently, temporal positional embedding is added to preserve temporal order:

(2) $$\left\{\begin{array}{c} TPE_{\left(pos,2i\right)}=\sin\left(pos/10000^{2i/d_{tpe}}\right)\\ TPE_{\left(pos,2i+1\right)}=\sin\left(pos/10000^{(2i+1)/d_{tpe}}\right) \end{array}\right.$$

where pos denotes the position index in the original sequence, and $d_{tpe}$ represents the embedding dimension. It is worth noting that to avoid introducing artificial bias, we did not employ any handcrafted task-specific or date-specific embeddings, thereby enhancing the generalization capability of the model.

After constructing the embedding representations, 75% of the subsequences are randomly masked, forming a masked subset $X_{mask}$ and an unmasked subset $X_{unmask}$, which can be formulated as:

(3) $$X_{unmask}=RandomMask(Conv1D(X_{hist})+PE(X_{hist}))$$

Only the unmasked subsequences are then fed into a stack of standard Transformer encoder blocks for deep contextual modelling and temporal dependency capture. Each encoder block consists of a multi-head self-attention (MHSA) layer and a feed-forward network (MLP), with residual connections and layer normalization:

(4) $$\left\{\begin{array}{c} {\hat{X}}_{unmask}=X_{unmask}+MHSA(LayerNorm(X_{unmask}))\\ {\tilde{X}}_{unmask}={\hat{X}}_{unmask}+MLP(LayerNorm({\hat{X}}_{unmask})) \end{array}\right.$$

where ${\tilde{X}}_{unmask}$ represents the contextual representation of the unmasked subsequences, serving as the preliminary encoding of trend features contained within the original time series.

#### 3.3.2. Pretext-Driven Sequence Reconstruction

To further strengthen the model’s capability in capturing long-term sequential trends, we designed a self-supervised prediction mechanism, which reconstructs the masked portions of the sequence using a Transformer decoder and a linear projection layer.

The decoder input consists of the contextual representations of unmasked subsequences ${\tilde{X}}_{unmask}$ and placeholder embeddings $X_{\left[mask\right]}$ for the masked positions. These are concatenated and passed through multiple Transformer decoder layers to generate a complete latent representation:

(5) $$\left\{\begin{array}{c} X_{hist}^{\prime }=Linear(Concat({\tilde{X}}_{unmask}),X_{\left[mask\right]})\\ \begin{array}{l} {\hat{X}}_{hist}=X_{hist}^{\prime }+MHSA(LayerNorm(X_{hist}^{\prime }))\\ {\tilde{X}}_{hist}={\hat{X}}_{hist}+MLP(LayerNorm({\hat{X}}_{hist})) \end{array} \end{array}\right.$$

Finally, a linear projection layer maps the decoder output back into the original input space to reconstruct the target sequence:

(6) $$X_{p\text{-}hist}=\mathrm{Projection}({\tilde{X}}_{hist})$$

During pre-training, the model is optimized by minimizing the mean absolute error (MAE) between the original sequence and its reconstruction. This objective serves as the core loss function, constraining the model to learn more accurate temporal representations.

Pre-training enables the model to internalize long-range temporal dependencies from large-scale historical data, capturing multi-day/weekly periodicity and slowly evolving trends as high-level temporal context. This context serves as a strong prior for forecasting: when inputs contain only short recent fragments, the pre-trained encoder supplies the missing long-term cues, improving the understanding of traffic dynamics and yielding more accurate, robust predictions. In essence, pre-training enhances sensitivity to long-range patterns and injects global context into short-term modeling, strengthening generalization under non-stationarity.

Processing full histories at inference is costly, so the prediction stage adopts a compute-efficient design: we used only the most recent subsequence as input, reflecting its highest relevance for near-term forecasting, while the encoder already parameterizes long-term knowledge learned during pre-training. After Conv1D and positional embedding, this latest subsequence is passed to the spatial graph convolution and convolutional trend-aware modules for further spatiotemporal extraction. This keeps inference lightweight while retaining the benefits of the pre-trained long-range temporal prior.

### 3.4. Dynamic Graph Learning

We modeled time-varying spatial dependencies with a dynamic graph learner parameterized by tensor decomposition. A learnable core tensor interacts with three sets of parameter matrices to generate an adjacency matrix for each time slice, enabling compact yet expressive modelling of complex, evolving spatial correlations. Unlike static-graph methods, the dynamic adjacency is updated end-to-end during training, allowing the graph structure to adapt continuously to non-stationary traffic patterns and improving downstream forecasting.

In practice, we assumed that traffic flows exhibit periodic regularities; that is, traffic conditions at the same temporal positions (e.g., morning or evening rush hours) tend to show similarity across different days. Based on this assumption, the dynamic graphs for identical time slots across days were shared, which reduces redundant computations and improves modelling efficiency [54,55]. Specifically, for each time slice, a corresponding dynamic graph was constructed, and all such graphs were organized into a three-dimensional adjacency tensor $A\in {\mathbb{R}}^{T\times N\times N}$, where T denotes the number of time slices and N represents the number of nodes in the road network. This design enables centralized storage and management of all dynamic graphs, while parameterization supports end-to-end optimization. Gradients are allowed to propagate through the learnable parameters within the tensor, ensuring that the dynamic graphs are iteratively refined and adaptively capture evolving traffic dependencies during training.

Moreover, the module is capable of reusing stable substructures within traffic networks. For instance, adjacent road segments typically exhibit strong correlations in flow, while commuting patterns between residential and commercial areas frequently reappear in daily cycles. Such recurring or spatially correlated structures can be effectively identified and preserved through the dynamic graph learning process, thereby enhancing the model’s ability to capture transferable patterns.

Formally, the adjacency tensor for the dynamic graphs can be expressed as:

(7) $$A=\mathrm{softmax}(\mathrm{ReLU}(E_{tc}\times E_{t}\times E_{sn}\times E_{tn}))$$

where $E_{t}\in {\mathbb{R}}^{T\times d_{G}}$ denotes the time-slot embedding, $E_{sn}\in {\mathbb{R}}^{N\times d_{G}}$ represents the source and $E_{tn}\in {\mathbb{R}}^{N\times d_{G}}$ target node encodings, $E_{tc}\in {\mathbb{R}}^{d_{G}\times d_{G}\times d_{G}}$ is the core tensor, and $d_{G}$ corresponds to the graph embedding dimension. Through this formulation, the resulting dynamic adjacency matrices can flexibly capture spatial dependencies across time slices, providing robust support for subsequent graph convolution operations. By retaining periodic traffic patterns while simultaneously adapting to evolving spatial structures, the dynamic graph learning module plays a critical role in modelling the complexity of traffic flow evolution and lays a solid foundation for improving overall prediction accuracy.

### 3.5. Spatial Graph Convolution

Real-world traffic exhibits tightly coupled, time-varying spatial and temporal interactions: flows evolve across time while the connections among road nodes change with conditions. High-accuracy forecasting therefore requires modelling continuously evolving dependencies in both space and time. Although prior work recognizes spatiotemporally, many methods rely on static spatial structures and cannot track how dependencies change over time, limiting adaptation to rapidly shifting patterns. We addressed this by prioritizing the temporal evolution of spatial dependencies and employing a dynamic graph convolution mechanism that updates the adjacency structure end-to-end, enabling the model to follow non-stationary traffic dynamics and improve predictive performance.

The core principle of spatial graph convolution is to aggregate the spatial information of a target node with the features of its neighboring nodes while leveraging a dynamic adjacency matrix to model the strength of their dependencies. Formally, at time t, the spatial representation of node i at the l-th layer can be computed as a weighted sum of the feature representations of nodes in its neighborhood N(i):

(8) $$H_{i,t}^{l}=\sum_{j\in N(i)}\alpha_{i,j}^{\left(t\right)}\cdot H_{j,t}^{l-1}$$

where $\alpha_{i,j}^{\left(t\right)}$ denotes the influence weight of node j on node i at time t, derived from the dynamic adjacency matrix learned in Section 3.4, and $H_{i,t}^{l}\in {\mathbb{R}}^{N\times d_{s.t}^{l}}$ represents the spatial embedding obtained from the l-th layer at time t. In other words, $\alpha_{i,j}^{\left(t\right)}$ encodes the time-varying spatial relationships, allowing node dependencies to be dynamically adjusted as time evolves. From a computational perspective, the spatial convolution can further be abstracted as a tensor–matrix multiplication:

(9) $$H_{S,t}^{l}=A^{\left(t\right)}\times H_{S.t}^{l-1}$$

where $H_{S,t}^{l}\in {\mathbb{R}}^{N\times d_{s,t}^{l}}$ represents the features of all nodes at the l layer and $A^{\left(t\right)}\in {\mathbb{R}}^{N\times N}$ is the dynamic adjacency matrix at time t. Finally, $H_{S}^{l}\in {\mathbb{R}}^{T\times N\times d_{s}^{l}}$ can be obtained by stacking $H_{S,t}^{l}$ along the time dimension. This tensorized formulation not only ensures efficient batch processing, but also enables the adjacency weights to be continuously optimized through gradient backpropagation, thereby achieving adaptive modelling of spatial dependencies as they evolve over time.

Through this mechanism, node representations are dynamically updated at each layer of the network, allowing the final predictions to simultaneously reflect both the local, real-time variations within spatial neighborhoods and the long-term evolutionary patterns of the global traffic network. By integrating such dynamic updates, the proposed method significantly enhances the ability to model complex spatiotemporal dependencies, thereby improving both predictive accuracy and generalization capability.

### 3.6. Convolutional Trend-Aware Attention

Temporal dependencies in traffic networks comprise both global correlations and local trends; neglecting either degrades forecasting. Unlike the RNN, LSTM, and CNN variants, the attention mechanism can directly capture long-range temporal relations without recursion, mitigating gradient vanishing and memory decay while adaptively emphasizing salient moments.

However, traffic flow data are typical continuous time series, where identical values may correspond to entirely different evolutionary phases. For instance, two equal flow values might occur under distinct conditions—one during a rapid growth phase and the other during a stable, low-variance period. Conventional attention mechanisms may in-correctly identify these two points as highly correlated, leading to biased temporal de-pendency modelling, reduced prediction accuracy, and insufficient capacity to capture local continuous trends.

To address this limitation, we introduced a convolutional trend-aware attention mechanism within the multi-head attention framework, inspired by convolutional attention [55]. The core idea is to incorporate a one-dimensional convolution when computing the Query (Q), Key (K), and Value (V) matrices, thereby aggregating contextual information from a neighborhood of time steps to explicitly encode local temporal trends:

(10) $$Q=\left(X*\phi^{Q}\right),K=\left(X*\phi^{K}\right),V=\left(X*\phi^{V}\right)$$

where ∗ denotes the convolution operation, and $\phi^{Q}$, $\phi^{K}$ and $\phi^{V}$ represent the convolution kernel parameters. Unlike traditional point-wise projections, this approach integrates local trend features into the representations of Q, K, and V.

The improved attention computation can then be formulated as:

(11) $$head_{i}=Attention(Q_{i},K_{i},V_{i})$$

(12) $$Q_{i}=\left(X*\phi_{i}^{Q}\right),K=\left(X*\phi_{i}^{K}\right),V=\left(X*\phi_{i}^{V}\right)$$

(13) $$Attention(Q,K,V)=\mathrm{softmax}\left(\frac{QK^{T}}{\sqrt{d_{h}}}\right)$$

(14) $$MHA_{trend}(Q,K,V)=Concat(head_{1},\ldots ,head_{h})W^{O}$$

where $d_{h}$ denotes the dimension of each attention head, h is the number of attention heads, and $W^{O}$ represents the learnable projection matrix.

This enhancement yields three benefits. First, it fuses global and local temporal dependencies by coupling convolution-based trend extraction with the global modelling capacity of multi-head attention. Second, it avoids spurious correlations by distinguishing timestamps that share the same value but follow divergent trends. Third, it strengthens continuity modelling, enabling the model to differentiate stable phases from volatile ones. Together, these effects produce more faithful temporal representations and improve robustness for dynamic traffic prediction.

### 3.7. Spatial and Temporal Feature Fusion

During the pre-training stage, the model acquires a strong ability to capture long-term dependencies and generates subsequence-level representations enriched with contextual information. Building upon this, we further utilized the embedding representation of the most recent subsequence as input. These embeddings were then fused with the spatial features extracted by the dynamic graph learning module and the temporal features derived from the convolutional trend-aware attention mechanism. The fusion is computed as follows:

(15) $$H_{F}=projection(H_{0})+MLP(H_{S}∥H_{T})$$

where $H^{0}\in {\mathbb{R}}^{T\times N\times d_{0}}$ represents the embedding obtained from the latest subsequence after the pre-training stage, $H_{S}\in {\mathbb{R}}^{T\times N\times d_{s}}$ denotes the embedding obtained by the spatial graph convolution at the last layer, $H_{T}\in {\mathbb{R}}^{T\times N\times d_{t}}$ denotes the embedding produced by the convolutional trend-aware attention module, projection(⋅) represents the semantic projection operation and ∥ denotes concatenation. This design offers two key advantages: on the one hand, it preserves the contextual knowledge learned during the pre-training stage; on the other hand, by jointly integrating spatial features, temporal dependencies, and global contextual information, it aligns and enhances the semantic space, thereby providing more discriminative representations for downstream prediction tasks.

### 3.8. Output Layer

The model employs fused feature representation to perform multi-step traffic flow prediction. Specifically, the forecasting results are obtained through a multi-layer perceptron (MLP) mapping, as follows:

(16) $$\hat{Y}=MLP(H_{F})$$

During training, the mean absolute error (MAE) is adopted as the objective function to jointly optimize the results across multiple prediction steps. This design not only ensures training stability, but also provides an effective measure of the deviation between the predicted and ground-truth values, thereby enhancing the overall performance of the model in multi-step forecasting scenarios.

## 4. Experiment

### 4.1. Dataset

To rigorously evaluate the effectiveness of the proposed method, we conducted experiments on four publicly available traffic datasets provided by the California Department of Transportation’s Performance Measurement System (PEMS). These datasets, collected from loop detectors embedded in freeways, capture real-time traffic operating conditions and are widely recognized as benchmark datasets in spatiotemporal prediction research [26].

The PEMS system aggregates data from more than 39,000 loop detectors deployed across major urban freeways in California, with the raw measurements recorded every 30 s. For both computational efficiency and practical relevance, the data were aggregated into 5 min intervals, resulting in 288 time steps per day. Missing values were imputed using linear interpolation to ensure continuity.

In this study, we focused on four geographically and temporally distinct subsets:

PEMS03: Collected from District 3, including 358 detectors, covering the period from 1 September to 30 November 2018.

PEMS04: Collected from the San Francisco Bay Area (District 4), containing 307 detectors, spanning 1 January to 28 February 2018.

PEMS07: Sampled from District 7, including 883 detectors, covering 1 May to 31 August 2017.

PEMS08: Obtained from the San Bernardino area (District 8), consisting of 170 detectors, covering 1 July to 31 August 2016.

A detailed summary of the statistical characteristics and key information of these four datasets is provided in Table 1.

### 4.2. Setting

All experiments were conducted on a computing platform equipped with an NVIDIA GeForce RTX 4090 GPU with 24 GB of memory. To ensure comparability with prior studies and maintain consistency with commonly adopted experimental setups, each dataset was partitioned chronologically into training, validation, and test sets with a ratio of 6:2:2. For the prediction task, the model uses the previous one hour of traffic flow data to forecast the following one hour, corresponding to an input and output window of 12 time steps.

During the pre-training stage, considering the larger scale and longer sequences of PEMS03 and PEMS07, one week of historical data was used as input, while for PEMS04 and PEMS08, two weeks of historical sequences were adopted to capture longer-term dependencies. Windows were sampled across all days in the training range (rather than a fixed few weeks) to avoid selection bias. Pre-training was conducted per-dataset, i.e., no cross-dataset or joint pre-training was performed. The masking ratio was 75% and exact subsequence length was two hours. The main hyperparameter settings in this stage include a latent hidden dimension of 96, a four-layer Transformer encoder, and a one-layer Transformer decoder, with each Transformer block configured with four attention heads.

In the subsequent prediction stage, only the most recent subsequence is fed to the predictor, while the long-term knowledge distilled in pre-training remains parameterized in the encoder and does not access the validation or test dataset. The batch size was set to 32, the maximum number of training epochs was 100, and the number of convolutional trend-aware attention heads was set to 8. Early stopping was applied based on validation performance to mitigate overfitting. The Adam optimizer was employed for training, while other key hyperparameters were fine-tuned individually for each dataset to achieve optimal model performance. Before inputting the data into our predictive model, we applied z-score normalization to standardize the data. Other details are shown in Table 2. This configuration not only ensures fairness and reproducibility when compared with existing methods, but also strikes a balance between computational efficiency and the ability to capture long-term temporal dependencies, thereby providing a stable and reliable environment for subsequent experiments.

### 4.3. Metric

We utilized three metrics to evaluate the predictive accuracy of the model during the experiments: Mean Absolute Error (MAE), Root Mean Square Error (RMSE), and Mean Absolute Percentage Error (MAPE). We also excluded missing values when calculating these metrics.

MAE is defined as follows:

(17) $$MAE=\frac{1}{N}\sum_{i=1}^{N}\left|y_{i}-{\hat{y}}_{i}\right|$$

RMSE is defined as follows:

(18) $$RMSE=\sqrt{\frac{1}{N}\sum_{i=1}^{N}{\left(y_{i}-{\hat{y}}_{i}\right)}^{2}}$$

MAPE is defined as follows:

(19) $$MAPE=\frac{1}{N}\sum_{i=1}^{N}\left|\frac{y_{i}-{\hat{y}}_{i}}{y_{i}}\right|\times 100\%$$

where $y_{i}$ is the true value and ${\hat{y}}_{i}$ is the prediction result.

### 4.4. Baselines

To comprehensively evaluate the performance of the proposed PT-TDGCN, we conducted comparative experiments against 14 baseline models, which were systematically categorized into three groups:

Traditional statistical and machine learning methods: including Historical Average (HA) [4], ARIMA [5], and Support Vector Regression (SVR) [7].

Spatiotemporal modelling approaches, which generally combine Graph Neural Networks (GNNs) with various temporal learning architectures: STGCN [23], DCRNN [24], STSGCN [26], ASTGCN [37], AGCRN [34], and DGCRN [40].

State-of-the-art advanced methods, which integrate cutting-edge techniques for enhanced spatiotemporal representation: Z-GCENETs [56], STGNCDE [57], DSTAGNN [41], NCSGCN [58], and TFormer [59].

### 4.5. Performance Result

PT-TDGCN consistently achieved a superior performance across all four PEMS datasets, as summarized in Table 3 and Table 4. Results in Table 3 and Table 4 are the mean over 5 seeds. For strong baselines (AGCRN, STGNCDE, TFormer), we also reported the mean ± std. Improvements over the strong non-ours baseline were statistically significant. The most remarkable improvements were observed on PEMS08, where the model reduced the MAE by 4.54%, RMSE by 5.61%, and MAPE by 3.83% compared to the second-best baseline. On the larger and more structurally complex PEMS07 dataset, PT-TDGCN still delivered stable gains, with reductions of 2.19% in MAE, 1.40% in RMSE, and 5.08% in MAPE. Similarly, continuous improvements were achieved on PEMS03 (MAE reduced by 3.99%) and PEMS04 (RMSE reduced by 3.25%). Despite significant differences in temporal coverage, node count, and spatial distribution among the four datasets, PT-TDGCN consistently maintained the highest prediction accuracy.

The stable gains arise from three design choices that distinguish PT-TDGCN from prior Transformer–GNN hybrids. First, a masked pre-training stage learns context-rich subsequence representations from long histories, strengthening long-range temporal dependency modelling. Second, a tensor-decomposition dynamic graph adaptively yields time-varying adjacency, overcoming static-graph rigidity and better tracking spatial evolution. Third, a convolutional trend-aware module captures local trend patterns while preserving global dependencies, improving sensitivity to regime changes without sacrificing context.

Comparative results show that traditional HA, ARIMA, and SVR are efficient and interpretable but limited by linear assumptions and fixed temporal structure, making them unsuitable for nonlinear, dynamic spatiotemporal patterns. Earlier STGNNs (e.g., STGCN, DCRNN, AGCRN) typically depend on static adjacency or fixed kernels, restricting adaptation to evolving spatial relations and long-range effects. More recent models with dynamic graphs (e.g., DGCRN, DSTAGNN) or Transformer-based designs (e.g., TFormer) improve flexibility but still fall short of jointly capturing local trend patterns and long-term temporal dependencies.

In contrast, PT-TDGCN closes these gaps by unifying masked pre-training, tensor-decomposition dynamic graphs, and convolutional trend-aware modelling, enabling simultaneous learning of long-term dependencies, adaptive spatial relations, and multi-scale temporal patterns. This synergy improves robustness to non-stationarity, periodicity, and abrupt fluctuations in traffic flow. Across four datasets, the results consistently validate PT-TDGCN’s higher accuracy and stronger resilience in real-world scenarios.

### 4.6. Ablation Study

To assess the efficacy of each component in PT-TDGCN, ablation experiments with 3 variants of our model were conducted, given as:

- *w*/*o* Pt. It removes the pre-training module;
- *w*/*o* DGL. It removes the dynamic graph learning module and replaced with a predefined graph;
- *w*/*o* ATT. It removes the a convolutional trend-aware attention module.

As shown in Table 5, Table 6, Table 7 and Table 8, the complete PT-TDGCN model consistently outperformed its ablated variants across all four PEMS datasets, confirming that each component makes a substantial contribution to overall performance. The ablation study yielded the following insights: (1) Pre-training module: removing the pre-training stage leads to noticeable performance degradation on all datasets, highlighting the critical role of subsequence-level representations for capturing long-term temporal dependencies. The most pronounced effect was observed on PEMS08, where MAE increased by 2.27% and MAPE by 2.20%. On PEMS04, MAPE showed the largest relative increase, accompanied by a 1.03% rise in RMSE. Although the absolute increments on PEMS03 and PEMS07 were relatively smaller, consistent deterioration was observed. On average, MAPE suffered the greatest increase across datasets, indicating that pre-training is particularly effective in reducing relative errors and enhancing robustness against scale variations and fluctuating patterns; (2) Dynamic Graph Learning module: replacing the dynamic graph learning module with a predefined static adjacency matrix result in consistent degradation across all datasets, validating the necessity of modelling time-varying spatial dependencies. The most significant impact appeared in the large-scale PEMS07 network, where MAPE increased by 1.66%. On PEMS03, the degradation was also clear, with MAE rising by 1.39%. These results suggest that static graphs are inherently inadequate for representing non-stationary correlations in real traffic networks, while tensor-decomposition-based dynamic graphs better align with evolving spatial structures across time slices; (3) Convolutional Trend-aware Attention module: excluding the convolutional trend-aware attention mechanism produced the largest degradation on PEMS03, with MAE increasing by 2.01% and MAPE by 1.44%. Similar deterioration was observed on PEMS07 and PEMS08. This demonstrates the importance of combining convolutional trend modelling with multi-head self-attention, which effectively suppresses noise, aligns local trends with global dependencies, and improves robustness under fluctuating traffic patterns. On average, the *w*/*o* ATT variant yielded the largest increase in MAE and RMSE across datasets, suggesting that trend-aware attention plays a more generalized role in reducing absolute errors and smoothing short-term volatility.

Overall, PEMS08 was the most sensitive to removing pre-training (long-term patterns), PEMS07 degraded most when replacing the dynamic graph with a static one (large-scale, complex spatial relations), and PEMS03 relied most on trend-aware modelling (short- to mid-term fluctuations). These dataset-specific sensitivities show that PT-TDGCN leverages the synergy of pre-training, dynamic graph learning, and convolutional trend-aware modelling to adapt across network scales and temporal regimes. Across all four public datasets, the complete model consistently attained the best MAE, RMSE, and MAPE, outperforming every ablated variant.

### 4.7. Computational Efficiency

To evaluate the parameter scale and computational efficiency of PT-TDGCN, we conducted experiments on the PEMS04 dataset, comparing it with four representative baseline models under identical hardware and training configurations. Among these baselines, DCRNN and AGCRN are classical traffic flow prediction methods: DCRNN excels at modelling temporal dynamics, while AGCRN learns node-specific spatiotemporal correlations without relying on predefined spatial graphs. Two advanced models with strong performance, STGNCDE and DSTAGNN, were also included for comparison: STGNCDE integrates GNNs with Neural Controlled Differential Equations (NCDE) to capture spatiotemporal dependencies, whereas DSTAGNN combines dynamic graph construction, spatiotemporal attention, and multi-receptive-field gated convolutions for dynamic spatiotemporal modelling.

Table 9 reports the parameter count of each model as well as the average training and inference time per epoch. It is important to note that pre-training is performed only once before the training stage and is therefore excluded from the runtime statistics. As shown in Table 9, PT-TDGCN contains more parameters than DCRNN, STGNCDE, and AGCRN but fewer than DSTAGNN, placing it at a mid-to-high parameter scale. In terms of runtime, PT-TDGCN requires 130.36 s/epoch for training and 24.69 s/epoch for inference, both higher than those of the four baselines. This suggests that the computational overhead arises not primarily from parameter size, but from operator complexity and constant factors: on the one hand, the Transformer architecture introduces higher computational density for temporal modelling; on the other hand, the dynamic graph learner incurs approximately $O(N^{2})$ level adjacency computation and feature aggregation at each time slice, particularly pronounced when adjacency matrices are dense. Furthermore, multi-branch feature fusion also adds extra parameters and computational cost.

In summary, PT-TDGCN achieves superior predictive accuracy at the expense of higher training and inference latency. This trade-off primarily stems from the collaborative design of pre-training, dynamic graph learning, convolutional trend-aware attention, and spatiotemporal fusion, which are precisely the components driving its consistent superiority across the four PEMS datasets. For practical deployment, the efficiency gap can be narrowed through engineering strategies such as: (1) adjacency sparsification prunes weak edges (or truncates low-rank factors) to reduce memory; (2) Top-K neighbor selection bounds per-node message passing by retaining only the highest-scoring dynamic neighbors; (3) time-slice caching reuses encoder states and time-varying adjacency within short horizons to amortize repeated computations; (4) lightweight attention mechanisms (local-window/dilated or block-sparse) replace full attention while preserving long-range cues via dilation; (5) mixed-precision training boosts throughput with negligible accuracy impact. These mechanism (sparsity level/Top-K, cache horizon K, window/dilation, precision) provide practical trade-offs between accuracy and efficiency without changing the core architecture.

### 4.8. Visualization Results

**Figure 2:**
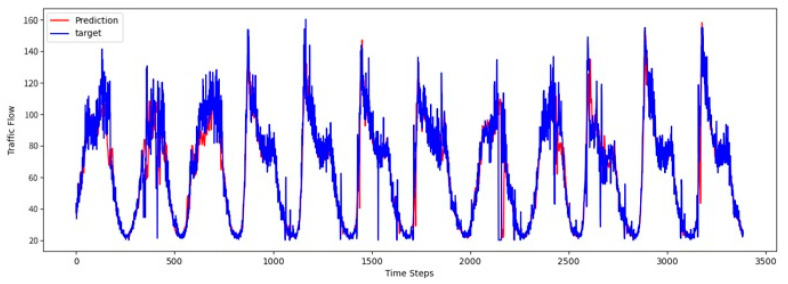
The visualization result on PEMS03.

**Figure 3:**
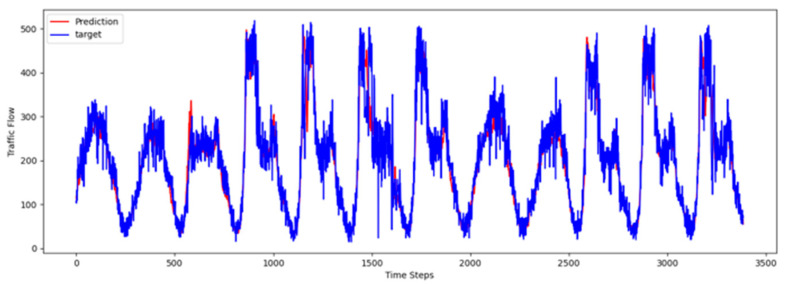
The visualization result on PEMS04.

**Figure 4:**
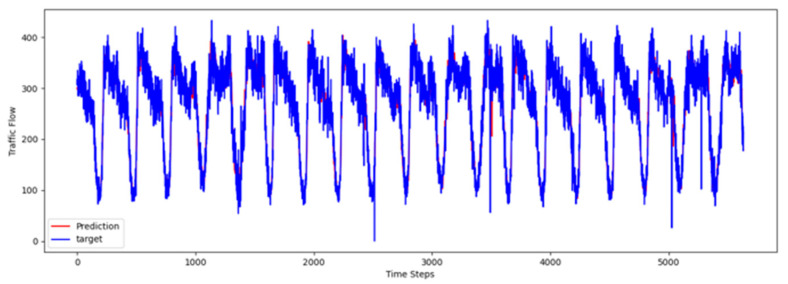
The visualization result on PEMS07.

**Figure 5:**
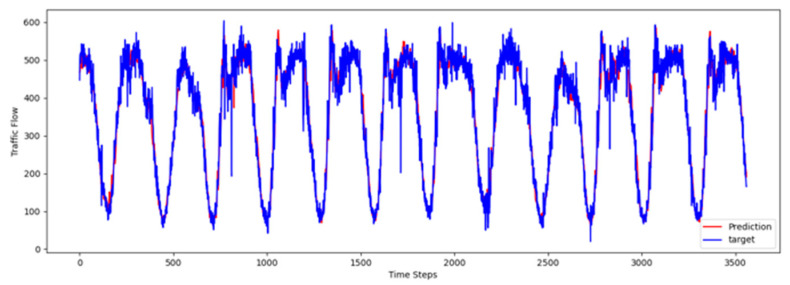
The visualization result on PEMS08.

To comprehensively validate the effectiveness of PT-TDGCN, we conducted visualization experiments on four datasets and compared the predicted results with the ground truth, as shown in Figure 2, Figure 3, Figure 4 and Figure 5. The results demonstrate that the model not only accurately reproduced the periodic temporal evolution of traffic states, but also maintained a sensitive and stable response to short-term fluctuations caused by non-routine scenarios such as holidays, rush hours, extreme weather, and unexpected incidents. During regular periods, the predicted peaks, troughs, amplitudes, and durations aligned closely with the actual curves, while in abnormal periods, the model promptly captured abrupt changes and effectively suppressed overshooting and lagging effects. From a spatial perspective, the tensor-decomposition-based dynamic graph learning adaptively updates adjacency relationships across time slices, enabling more precise characterization of congestion propagation paths and delay alignment within the network. Meanwhile, the convolutional trend-aware attention explicitly encodes local temporal trends in the Q/K/V projections and synergizes them with global dependencies captured by multi-head attention, thereby balancing short-window fluctuations and overall patterns. Furthermore, the long-term contextual representations obtained during the pre-training stage provide robust priors for abnormal scenarios, enhancing generalization and resilience. Cross-region and cross-scale comparisons further confirm that PT-TDGCN achieved high consistency with real-world trajectories in both global patterns and local details, demonstrating its robustness and adaptability when facing complex and non-stationary traffic dynamics.

## 5. Conclusions

We propose PT-TDGCN, a unified framework that integrates pre-trained long-term dependencies, dynamic spatial relationships, and convolutional trend-aware attention to systematically address three key challenges in traffic prediction: the limitations of static graphs, the difficulty of capturing global temporal correlations, and the inability to balance long-term dependencies with short- to mid-term trends. By performing masked subsequence pre-training on extended historical sequences, the model learns context-rich long-term representations. Combined with tensor-decomposition-based dynamic graph learning and convolutional trend-aware attention, PT-TDGCN jointly captures time-varying spatial dependencies and local temporal patterns, which are further fused with spatial graph convolution and lightweight integration for end-to-end prediction. Extensive experiments conducted on four public datasets demonstrated that PT-TDGCN consistently outperformed 14 baseline models. Ablation studies further confirmed the complementary roles and necessity of each component. Our proposed framework can be applicable to common traffic-sensing settings and shows clear potential for integration within intelligent transportation systems (ITS), where its modular components can interface with standard data feeds and operational workflows to provide reliable short-horizon forecasts for decision support.

There are also several limitations to this study: our evaluation relied on PEMS benchmarks; practitioner-facing interpretive analysis is limited; and under full settings, the model may incur a higher runtime than lighter baselines. In the future, we plan to enhance PT-TDGCN in five directions: (1) systematically incorporate external variables such as weather, holidays, sports events, roadwork, and accidents to construct event-triggered dynamic priors and improve adaptability to non-stationary scenarios; (2) integrating multimodal signals including trajectories, images, text, and social events to achieve more comprehensive traffic state awareness; and (3) extending prediction horizons to multi-hour, cross-day, and even cross-week settings through multi-scale temporal pyramids and hierarchical decoders to explicitly model periodicity and drift; (4) extending the interpretability component by adding practitioner-oriented visual summaries (e.g., spatial heatmap-style summaries from dynamic adjacency and temporally aggregated channel contributions) to provide more explanatory insights without altering the core architecture; (5) expanding evaluation to additional real-world datasets beyond PEMS (e.g., Asia and Europe networks) to more rigorously assess generalizability and robustness. Through these efforts, we aim to evolve PT-TDGCN from a short-term high-accuracy predictor into a unified spatiotemporal framework that balances long-term stability, cross-domain generalization, and practical deployability, thereby enhancing its application value in real-world traffic systems.

## Figures and Tables

**Table 1:** The description of datasets.

Dataset	#Sensor	#Edges	#Time Steps	#Time Interval	Time Range
PeMS03	358	547	26,208	5 min	1 September 2018–30 November 2018
PeMS04	307	340	16,992	5 min	1 January 2018–28 February 2018
PeMS07	883	866	28,224	5 min	1 May 2017–31 August 2017
PeMS08	170	295	17,856	5 min	1 July 2016–31 August 2016

**Table 2:** The other training settings.

Item	Value
Adam betas Weight decay	0.9, 0.999 1 × 10^−4^
Learning rate	5 × 10^−4^
Gradient clipping	1.0
Early stopping patience	15 epochs/Val MAE

**Table 3:** The performance results on PEMS03 and PEMS04.

Model	PEMS03			PEMS04		
	MAE	RMSE	MAPE	MAE	RMSE	MAPE
HA	31.72	52.43	33.88	38.12	59.63	27.95
ARIMA	35.62	48.02	33.92	33.62	48.92	24.63
SVR	21.99	35.43	21.62	28.82	44.58	19.23
STGCN	17.53	30.49	17.26	21.22	34.95	13.92
DCRNN	18.05	30.34	18.42	21.24	33.49	14.19
STSGCN	17.52	29.25	16.93	21.25	33.79	13.99
ASTGCN	17.39	29.59	17.19	22.97	35.31	16.58
AGCRN	15.98 ± 0.23	28.25 ± 0.14	15.26 ± 0.15	19.84 ± 0.14	32.32 ± 0.10	12.96 ± 0.11
DGCRN	15.97	27.43	17.76	20.42	32.39	14.67
Z-GCENETs	16.68	28.17	16.42	19.53	31.64	12.83
STGNCDE	15.62 ± 0.18	27.13 ± 0.12	15.09 ± 0.10	19.23 ± 0.12	31.12 ± 0.09	12.77 ± 0.13
DSTAGNN	15.59	27.25	14.68	19.30	31.49	12.70
NCSGCN	15.33	24.98	16.52	20.34	33.49	14.58
TFormer	15.03 ± 0.13	25.31 ± 0.12	15.37 ± 0.11	18.94 ± 0.14	31.36 ± 0.12	12.72 ± 0.09
PT-TDGCN	**14.43 ± 0.08**	**24.72 ± 0.05**	**14.61 ± 0.05**	**18.62 ± 0.10**	**30.11 ± 0.05**	**12.61 ± 0.02**

**Table 4:** The performance results on PEMS07 and PEMS08.

Model	PEMS07			PEMS08		
	MAE	RMSE	MAPE	MAE	RMSE	MAPE
HA	45.22	65.72	24.53	34.88	59.31	27.92
ARIMA	38.19	59.33	19.49	31.22	44.35	22.87
SVR	32.51	50.27	14.26	23.31	36.18	14.67
STGCN	25.31	39.42	11.23	17.58	27.13	11.31
DCRNN	25.24	38.63	11.87	16.85	26.39	10.92
STSGCN	24.26	39.03	10.21	17.16	26.85	10.99
ASTGCN	24.03	37.88	10.76	18.22	28.09	11.76
AGCRN	22.39 ± 0.15	36.61 ± 0.23	9.16 ± 0.18	15.99 ± 0.17	25.25 ± 0.14	10.13 ± 0.10
DGCRN	20.56	33.59	9.12	16.25	26.18	12.07
Z-GCENETs	21.79	35.17	9.28	15.78	25.13	10.09
STGNCDE	20.59 ± 0.14	33.86 ± 0.25	8.86 ± 0.15	15.47 ± 0.14	24.83 ± 0.09	9.92 ± 0.12
DSTAGNN	21.45	34.53	9.03	15.69	24.77	9.97
NCSGCN	20.98	34.71	10.05	17.69	27.09	10.58
TFormer	20.77 ± 0.16	34.07 ± 0.18	8.92 ± 0.12	15.21 ± 0.12	24.89 ± 0.10	9.93 ± 0.09
PT-TDGCN	**20.11 ± 0.12**	**33.12 ± 0.12**	**8.41 ± 0.08**	**14.52 ± 0.10**	**23.38 ± 0.08**	**9.54 ± 0.03**

**Table 5:** The ablation study on PEMS03.

Dataset	Model	MAE	RMSE	MAPE
PEMS03	*w*/*o* Pt	14.56	24.79	14.78
	*w*/*o* DGL	14.63	24.82	14.75
	*w*/*o* ATT	14.72	24.91	14.82
	PT-TDGCN	**14.43**	**24.72**	**14.61**

**Table 6:** The ablation study on PEMS04.

Dataset	Model	MAE	RMSE	MAPE
PEMS04	*w*/*o* Pt	18.71	30.42	12.83
	*w*/*o* DGL	18.77	30.25	12.69
	*w*/*o* ATT	18.84	30.37	12.76
	PT-TDGCN	**18.62**	**30.11**	**12.61**

**Table 7:** The ablation study on PEMS07.

Dataset	Model	MAE	RMSE	MAPE
PEMS07	*w*/*o* Pt	20.19	33.22	8.48
	*w*/*o* DGL	20.22	33.27	8.55
	*w*/*o* ATT	20.28	33.35	8.51
	PT-TDGCN	**20.11**	**33.12**	**8.41**

**Table 8:** The ablation study on PEMS08.

Dataset	Model	MAE	RMSE	MAPE
PEMS08	*w*/*o* Pt	14.85	23.47	9.75
	*w*/*o* DGL	14.67	23.42	9.61
	*w*/*o* ATT	14.72	23.52	9.68
	PT-TDGCN	**14.52**	**23.38**	**9.54**

**Table 9:** The computational efficiency results.

Model	Parameters	Training (s/epoch)	Inference (s/epoch)
DCRNN	298,049	36.35	3.28
AGCRN	748,810	16.92	1.84
STGNCDE	322,588	67.92	8.55
DSTAGNN	3,579,728	100.23	11.21
PT-TDGCN	1,264,672	130.36	24.69

## Data Availability

The data used in the experiments of this work are included or references are provided in the article.
